# Photo Quiz: Unexpected finding in a Gram stain

**DOI:** 10.1128/jcm.00641-23

**Published:** 2023-11-21

**Authors:** Cristina Bayo Sánchez, Iosu Razquin Olazarán, Carmen Martin Salas, Maria Eugenia Portillo Bordonabe, Carmen Ezpeleta Baquedano

**Affiliations:** 1 Servicio de Microbiología. Hospital Universitario de Navarra, Pamplona, Navarra, Spain; Mayo Clinic Minnesota, Rochester, Minnesota, USA

**Keywords:** *Strongyloides*, disseminated infection, Gram stain

## PHOTO QUIZ

### ­

**Fig 1:**
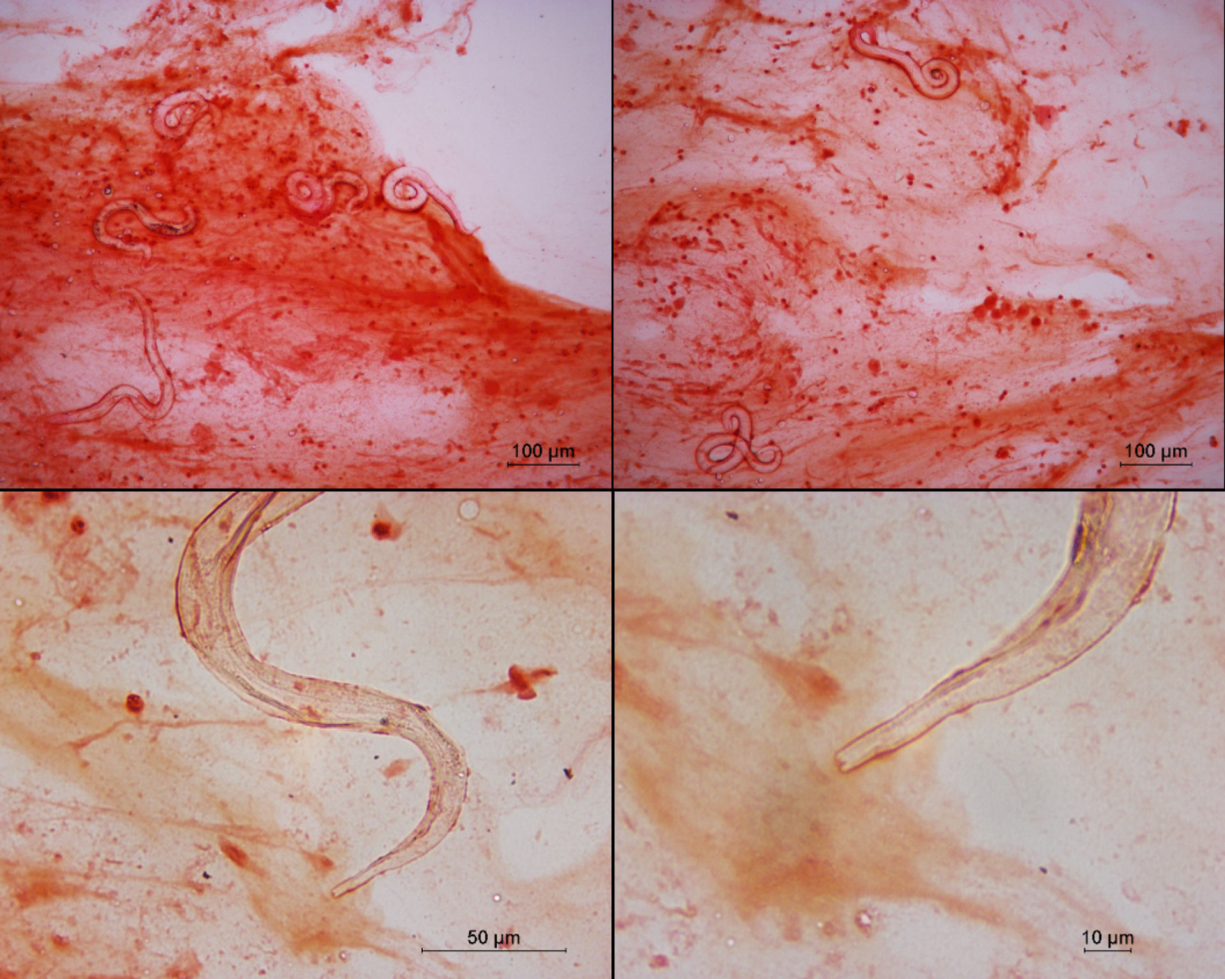
(A/B) Low-power view (×100 magnification) of Gram stain of larvae in tracheal aspirate showing multiple autoinfective filariform (L3a) larvae of *Strongyloides stercoralis*. (C/D) Notched tail of autoinfective filariform (L3a) larvae of *Strongyloides stercoralis* (×400 and ×1000 magnification, respectively).

A 60-year-old Ecuadorian man living in Spain since 1999, with intermittent peripheral eosinophilia of unknown cause from 2013 to 2017, was admitted to the hospital with a 1-month history of subdural hematoma after a traffic accident. In previous consultations, he reported neck pain, intense headache, paresthesias in the right arm, and dizziness, and he was treated with dexamethasone. In the current episode, he presented with 24-h abdominal pain, loose stool, and vomiting, without neurological symptoms. He had sinus tachycardia (115 bpm), and the initial laboratory test revealed leukocytosis (18,300 leukocytes/mL) without eosinophilia, increased acute phase reactants (C-reactive protein of 123 mg/L), and elevated lactate (6.6 mmol/L). Computed tomography demonstrated non-specific ileitis with possible bowel ischemia, but he was discharged again with dexamethasone and analgesics. The next day, he returned with worsening general condition, tachycardia, hypotension, and desaturation, as well as a higher C-reactive protein than the previous day (238 mg/L). Subsequently, empirical intravenous 4 g/24 h piperacillin-tazobactam was initiated. After an ileocecal resection by laparotomy, he developed septic shock and multiple organ failure. Two sets of blood cultures, urine, and tracheal aspirate specimens were collected. *Escherichia coli*, resistant to ampicillin but susceptible to all antibiotics tested, grew in blood cultures. Gram stain of a tracheal aspirate showed objects identified as helminth larvae (Fig. 1A through D). Due to these findings, 16 mg/24 h ivermectin and 400 mg/12 h albendazole were added to the treatment. Additionally, physical examination revealed petechial and purpuric patches in the lower trunk, so cutaneous biopsies were obtained. Ascitic fluid and stool specimens were also collected.

